# Effectiveness of Toki’s criteria and determination of variables for identification of HPV L1 protein in oral lesions

**DOI:** 10.4317/medoral.19748

**Published:** 2014-06-01

**Authors:** Josué R. Bermeo-Escalona, Blanca S. González-López, Eva Ramón-Gallegos, Hugo Mendieta-Zerón

**Affiliations:** 1 C.D. Doctorate Student in Health Sciences. Faculty of Medicine, Universidad Autónoma del Estado de México (UAEMex), Toluca, México; 2 Doctorate in Dentistry. Chief of the Oral Pathology Laboratory. Centro de Investigación y Estudios Avanzados en Odontología (CIEAO), UAEMex, Toluca, México; 3 Doctorate in Chemical-Biological Sciences. Chief of the Environmental Cytopathology Laboratory. Escuela Nacional de Ciencias Biológicas (ENCB), Instituto Politécnico Nacional (IPN), México, D.F; 4 PhD in Endocrinology. Chief of the Laboratory of Molecular Biology. Centro de Investigación en Ciencias Médicas (CICMED), UAEMex, Toluca, México

## Abstract

Objectives: To evaluate the effectiveness of Toki’s criteria in identifying the HPV L1 protein in oral lesions with the use of immunohistochemistry (IHC) and to determine which criteria optimize such identification. Study Design: Retrospective study of 277 cases diagnosed as HPV lesions at 22 years. Tests of sensitivity, specificity, positive predictive value (PPV), negative predictive value (NPV), kappa coefficients, and chi2 values, as well as two logistic regression analyses (p≤0.05), were conducted. Results: Of the lesions studied, 96.4% (267 of 277) were positive for HPV using Toki’s criteria and 28.5% (79 of 277) were positive for L1 by IHC. Toki’s criteria showed sensitivity=93.67%, specificity=2.53%, PPV=6.99%, and NPV=46.55%. Neither concordance nor statistically significant associations were observed between both tests. The logistic regression of Toki’s criteria was useful in the diagnosis of L1, correctly classified 71.8% of the lesions positive for L1, and showed a Hosmer-Lemeshow adjustment of *p*=0.614 and a Nagelkerke’s coefficient of determination of 6.8%. The explanatory variables statistically significant at *p*≤0.05 were dyskeratosis (*p*=0.01) and papillomatosis (*p*=0.04). Forty-nine independent variables (clinical and histopathologic) were involved in the second regression analysis. The model correctly classified 85.2% of the lesions and showed a Hosmer-Lemeshow adjustment of *p*=0.696 and a Nagelkerke’s coefficient of determination of 60.2%. The explanatory variables statistically significant at*p*≤0.05 were: age younger than 35 years (*p*=0.001), multiple lesions (*p*=0.031), hyperorthokeratosis (*p*=0.019), focal intracellular edema (*p*=0.002), and the presence of 1 to more than 5 cells with degenerative changes in their nucleus (*p*=0.048). Conclusions: Toki’s criteria are not adequate to make a diagnosis of lesions by HPV in the mouth, but the logistic regression analysis showed clinical and histopathologic variables which optimize the identification of lesions through the L1 protein. However, a PCR study is advisable when the presence of high-risk HPV is suspected.

** Key words:**HPV, Toki’s criteria.

## Introduction

The human papillomavirus (HPV) is a member of the papillomaviridae family (1), which can infect the oral mucosa of adults and children by different modes of transmission (2,3). This infection takes place in the cells of the basal layer and over a variable incubation period of up to 20 years, which may culminate in a latent, subclinical, or clinical infection where benign, potentially malignant, or malignant lesions can become evident (4).

In 1987, Toki defined the use of a scoring system called “HPV score” for detecting HPV in cervical lesions. The system consists of giving a score on six histologic findings: koilocytosis, 4; bi- and multinucleation, 2; dyskeratosis, 1; intraepithelial capillary loops (papillomatosis), 1; basal cell hyperplasia, 1; and acanthosis, 1. The maximum score using this system is 10 points, and a diagnosis of HPV is declared when a score of over 6 points is achieved (5). However, the molecular behavior of the virus in the mouth is different from that in the cervix and as subclinical as clinical infection independently of their type can present its genome as episomal, integrated, or both (6), even in events of oral carcinoma (OC) (7).

The HPV contains 7,200 to 8,000bp in a circular double-stranded DNA molecule covered by a capsid of proteins of icosahedral form with 72 capsomeres (6). Their DNA encodes eight open reading frames (ORFs), six early (E) ORFs (E1, E2, E4, E5, E6, and E7) that encode nonstructural regulatory proteins, and two late (L) ORFs (L1 and L2) that encode proteins for the capsid. In addition, the HPV genomes have a region called the upstream regulatory region (URR); this is located between the early and late regions and includes the origin of the replication, the E6/E7 promoter gene, enhancers, and silencers (6).

The expression of early genes occurs throughout all epithelial layers (4). The E1 and E2 proteins surround the origin of the viral replication and are responsible for maintaining the viral DNA as episomes. In particular, E2 has the ability to suppress the activity of the E6 and E7 promoters (8).

In infections of high risk, the integration of the viral DNA into the cell genome alters the E2 ORF, which causes the loss of the repressive function of the E2 protein, increasing the expression of the E6 and E7 oncoproteins (8). This facilitates the cell-to-cell replication of the DNA virus and the decrease in the expression of L1 (9). On the other hand, in infections of low risk, the E1 protein recruits the cell elements necessary for the episomal replication, and the expression of late genes occurs in the keratinocytes differentiated in the upper layers, where the assembly of the viral capsids take place, leading to the formation of virions (4). The L1 protein is expressed after the L2 protein in the viral replication cycle; it is commonly used as an epitope in immunohistochemistry (IHC), whereas the L2 protein interacts with the E2 protein, facilitating the transport of the L1 protein to the nucleus (9).

The objectives of this study are to assess the efficacy of Toki’s criteria in identifying through IHC the presence of HPV L1 protein in oral mucosal lesions by HPV and to determine the clinical and histopathologic variables that optimize such identification.

## Material and Methods

This is a retrospective and descriptive study of 277 biopsies fixed in 4% formaldehyde buffer, embedded in paraffin, preserved at ambient temperature, and previously diagnosed through histopathologic study as lesions by HPV over a period of 22 years (1990-2012). Lesions with diagnosis of multifocal epithelial hyperplasia (MEH), verruca vulgaris, and condyloma acuminatum, which are considered as lesions by HPV (10,11) were included in the study, while 99/376 lesions under inefficient fixation conditions, not fixed in 4% formaldehyde buffer, with insufficient material or those without conclusive characteristic of lesion by HPV were excluded.

The diagnoses were established with the use of clinical and morphologic criteria. Unique or multiple, small, white or pink papular lesions featuring sessile elevations which may show finger-like projections of stratified squamous epithelium with wide variations in the thickness of the keratin layer, usually parakeratinized and koilocytosis microscopically (10,11).

The clinical characteristics were obtained from the histopathologic applications of patients, and the morphologic data were gathered from the observation of cuts on slides with the use of an Olympus optical microscope. To determine the presence of HPV L1, tissues were stained by IHC.

Forty-nine independent variables consisting of 15 clinical and 34 histopathologic characteristics categorized as structural or cellular variables were evaluated.

- Clinical variables: Age and gender of the patient, and location, number, contour, base, surface, consistency, color, type, edge, mobility, pain, diameter, and evolution of lesions.

- Structural variables: The presence of papillomatosis, pseudoepitheliomatous hyperplasia, acanthosis, and a granular layer (light, moderate, or prominent); the presence and intensity of hyperparakeratosis, hyperorthokeratosis, or both keratins (considering only the intensity of the hyperkeratosis presented); epithelial hyperplasia, inflammatory infiltrate, and basal cell hyperplasia; and the presence, intensity, and disposition of both intracellular and extracellular edema were evaluated.

- Cellular variables: The presence of dyskeratosis, pinocytosis, keratin pearls, and pleomorphism; the presence and number per field of cells with degenerative changes in their nucleus, binucleated cells, and mitosis; and the presence, intensity, disposition, and layer of the koilocytosis were evaluated.

The intensity of the histologic characteristics was registered as light, moderate, or severe, and the disposition as isolated, focal, or generalized; the cellular changes and mitosis were counted by examining all fields.

- Toki’s criteria: Acanthosis, “1”; dyskeratosis, “1”; basilar hyperplasia, “1”; koilocytosis, “4”; bi- or multinucleated cells, “2”; and papillomatosis, “1” (5).

- Immunohistochemistry: Each of the lesions was cut to 10µm for IHC staining for HPV; monoclonal mouse anti-human papillomavirus (SB24) detects the epitope L1. Deparaffinized sections were immersed in absolute methanol containing 0.03% (v/v) H2O2 at room temperature to block endogenous peroxidase activity. After washing with phosphate-buffered saline (1x PBS, pH 7.4), the sections were immersed in 0.01M citrate buffer (pH 6.0) and heated in a pressure cooker for 15 minutes. The sections were incubated in 2% (w/v) bovine serum albumin in 1x PBS for 15 minutes at room temperature to block nonspecific reactions.

Appropriately diluted HPV antibody (1:100) was applied to each section for 30 minutes at room temperature. After washing with 1x PBS, the sections were incubated with IgM biotinylated goat anti-rabbit (H+L) antibody (1:200, Vector Laboratories Inc.) for 10 minutes at room temperature and then washed with 1x PBS. Appropriately diluted streptavidin-peroxidase (1:200, Gibco-BRL, Grand Island, NY, USA) was applied to each section for 10 minutes. Then the sections were again washed with 1x PBS, immersed for 3 minutes in 0.05% 3,3’-diaminobenzidine tetrahydrochloride in 0.05M Tris-HCl buffer (pH 7.6) containing 0.01% (v/v) H2O2 (DAB), and counterstained with Mayer´s hematoxylin. Lesions with reaction intensity, light, moderate or severe with a disposition isolated, focal or generalized in the cell nuclei of the spinous layer were considered positive to HPV L1.

- Statistical analysis: The values of the lesions according to Toki’s criteria, the clinical and morphologic characteristics, and the positivity for L1 were registered in a data base in the SPSS 20 software (IBM, NY, USA). To evaluate the effectiveness of Toki’s criteria in the diagnosis of HPV L1 through IHC, tests of sensitivity, specificity, positive predictive value (PPV), and negative predictive value (NPV) with the use of Bayes’ theorem were developed. The association and concordance between the diagnosis of HPV using Toki’s criteria and the identification of the L1 protein by IHC were calculated by the “chi2” and kappa coefficients, considering the valuation of Landis and Koch (12). Two logistic regression analyses were done, one to determine which Toki’s criteria are useful for the diagnosis of HPV L1 in the mouth and another to determine the clinical and histopathologic criteria that optimize such diagnosis. In the first regression analysis, the presence of HPV L1 as determined by IHC was considered as the dependent variable and Toki’s criteria as independent variables; in the second, all the clinical and histopathologic variables were evaluated. The categorical variables were transformed into dummy variables in each case, the reference categories were chosen, and the method used was “Enter”. The adjustment of the models was assessed by the Hosmer-Lemeshow test and the Nagelkerke’s coefficient of determination. The statistically significant coefficients (*p*≤0.05), odds ratio (OR), and confidence intervals at 95% were obtained, and receiver operating characteristic (ROC) curves were constructed.

## Results

During the 22-year period considered, 5,037 studies were done in the laboratory, and 277 cases were diagnosed as lesions by HPV in the mouth, presenting a prevalence of 5.49%.

The relation between the age range of patients and their gender are show in  Table 1, while the main clinic characteristics reported are show in  Table 2. Although some habits as smoking and drinking are included in the histopathologic applications of patients, often they are not reported by clinicians, therefore they could not be assessed in this study.

**Table 1:**
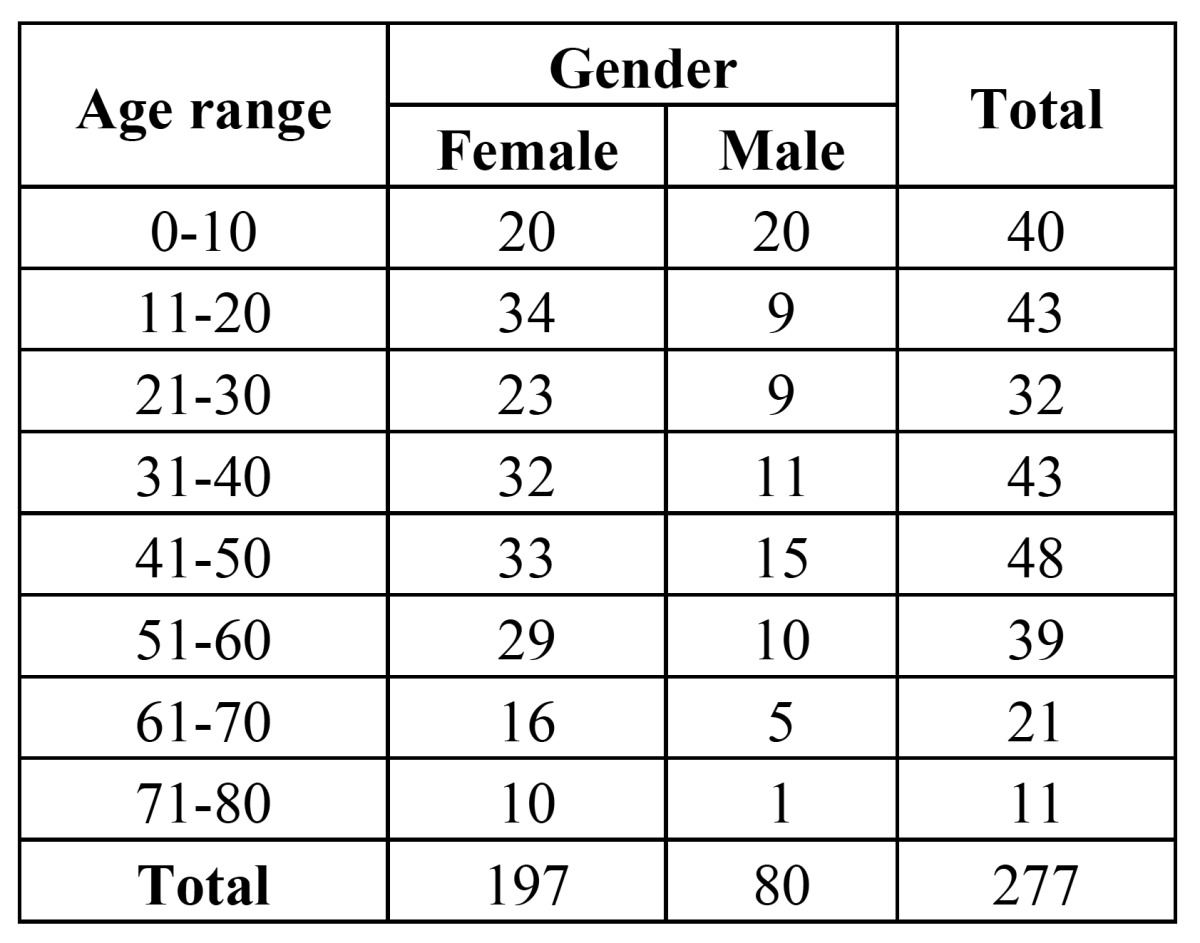
Relation between the age range and gender of patients.

**Table 2:**
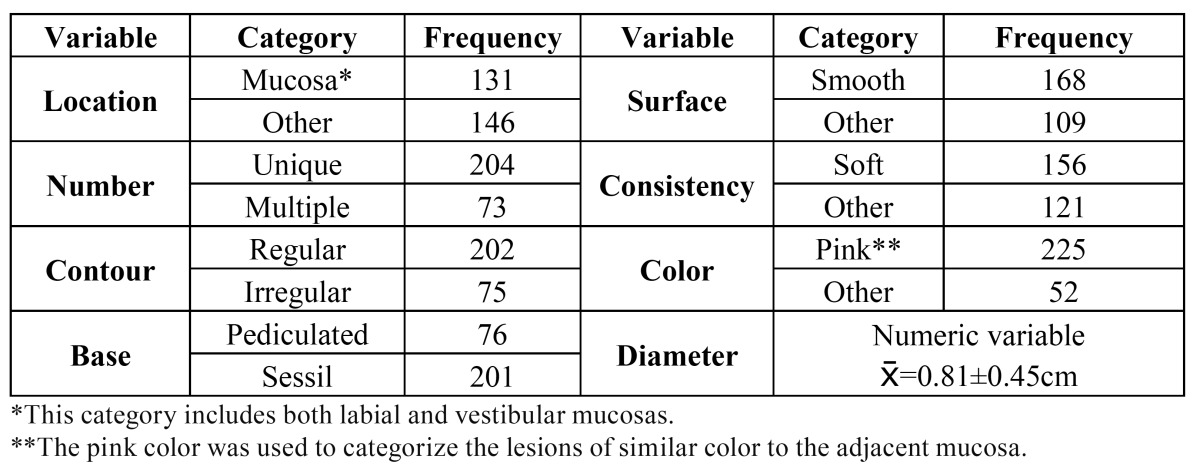
Main clinic characteristics of lesions reported by clinicians.

- Evaluation of Toki’s criteria for the diagnosis of lesions by HPV in the mouth through the identification of L1 by IHC.

The 96.4% (267 of 277) of lesions had scores greater than or equal to 6 when Toki’s criteria were used, whereas only 28.5% (79 of 277) were found to be positive for L1 by IHC. When compared with the IHC test to identify HPV L1, the Toki’s criteria showed a sensitivity of 93.67%, a specificity of 2.53%, a PPV of 6.99%, and a NPV of 46.55%. Neither a significant association (chi2 *p*=0.155) nor a statistical concordance (kappa coefficient *p*=0.125) was found between Toki’s criteria and the IHC test for L1.

The logistic regression model developed to identify which of the 6 Toki’s criteria are useful in the diagnosis of L1 by IHC correctly classified 71.8% of the lesions positive for HPV L1, and showed a Hosmer-Lemeshow adjustment of *p*=0.614 and a Nagelkerke’s coefficient of determination of 6.8%.  Table 3 shows the Toki’s criteria that enable the diagnosis of HPV by IHC using the epitope L1. The koilocytosis did not show statistical significance but was retained because it supported the adjustment of the model. The ROC curve for the Toki’s criteria shows that they have regular diagnostic value, with an area under the curve (AUC) of 0.632 (*p*=0.001). Nevertheless, the cutoff for a sensitivity of 87.3% shows a specificity of 39.8% and a probability of false positives of 60.2% when Toki’s criteria are used in the diagnosis of HPV lesions in the mouth where there is L1 expression.

**Table 3:**
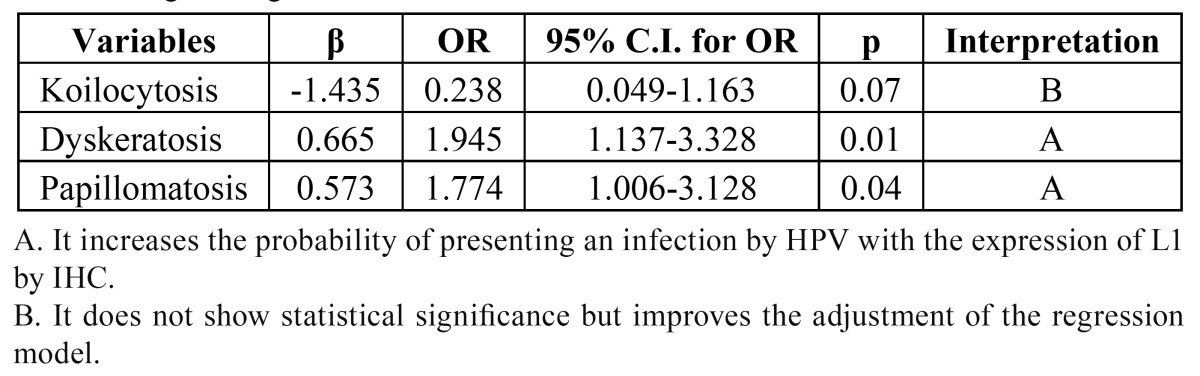
Logistic regression of Toki’s criteria.

- Evaluation of clinical and histopathologic variables for the diagnosis of lesions by HPV in the mouth through the identification of L1 by IHC.

The logistic regression model developed correctly classified 85.2% of the lesions positive for HPV L1 infection, and showed a Hosmer-Lemeshow adjustment of *p*=0.696 and a Nagelkerke’s coefficient of determination of 60.2%. The final model included 20 variables, namely, 6 clinical, 10 structural, and 4 cellular variables, which improved its adjustment: The clinical variables were age of patient, and number, contour, base, surface, and location of the lesions. The structural variables included hyperorthokeratosis, pseudoepitheliomatous hyperplasia, papillomatosis, and granular layer; intensity of the epithelial hyperplasia, hyperkeratosis, and extracellular edema; presence and intensity of the inflammatory infiltrate; and disposition of the intracellular edema. The cellular variables were presence of pinocytosis, presence and number of cells with degenerative changes in their nucleus (1 to 4 per field, up to 5 per field, and more than 5 per field), and layer of the koilocytosis.

Nevertheless, only 5 of these variables presented a level of significance of *p*≤0.05. These included age, with patients older of 35±20.2 years having decreased probability of infection by HPV associated with the expression of L1 by IHC; presence of multiple lesions, hyperorthokeratosis, and focal intracellular edema, which increase the risk of infection; and the presence of cells with degenerative changes in their nucleus, which is associated with the infection in general but showed no relevant data in the categories ( Table 4).

**Table 4:**
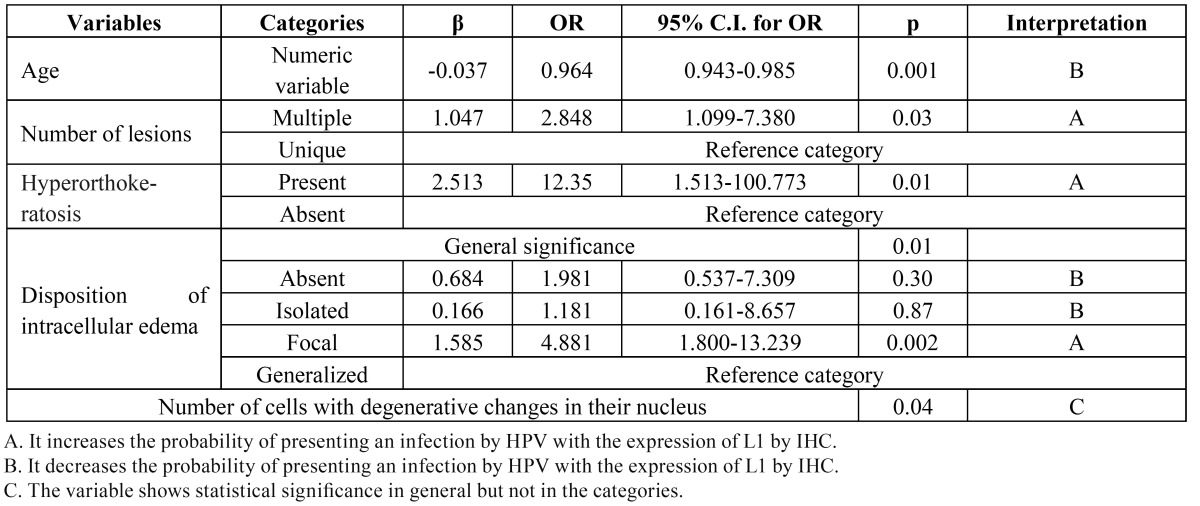
Logistic regression of the clinical and histopathologic variables.

The rest of the variables presented significance levels of *p*>0.05 but were kept in the model to improve its adjustment (data not shown). The ROC curve for the clinical and histopathologic variables shows that they have a very good diagnostic value (AUC=0.912, *p*<0.001). The cutoff for a sensitivity of 81% shows a specificity of 85.9% and a probability of false positives of 14.1% when these criteria are used in the diagnosis of HPV lesions in the mouth where there is L1 expression.

## Discussion

The prevalence of OC has increased in the last decade, and because HPV is the most important biological factor for the disease, the proper diagnosis of HPV is crucial (13).

Toki’s criteria are used for the diagnosis of cervical HPV, and due to the simplicity of the system, its application has been extended to the field of dentistry (14). However, the results of this study imply that its use may be inadequate in oral lesions. The percentages of sensitivity, specificity, and PPV presented by Toki’s criteria suggest a high probability of false positives when used in the diagnosis of lesions by HPV in the mouth associated with the expression of L1 by IHC.

HPV in the mouth sometimes does not behave as theoretically expected, and the HPVs 16 and 18, prevalent in potentially malignant oral lesions, typically show a low viral load and rarely integrate into the cell genome (15,16). In fact, the viral genome in the lesions of oral mucosa is generally found in its episomal form, even in OC, where the tests of integration of the viral DNA are usually negative, this suggests that the model for cervical cancer is inapplicable to oral tumors (16).

Due to the low number of viral copies that have been found in carcinomas and high-risk lesions, the “hit and run” theory has been developed, which proposes that tumors negative for HPV could develop a precursor with HPV content without requiring the HPV to remain in the malignant state (17).

On the other hand, the IHC tests used for detection of HPV usually use the structural protein L1 as epitope; however, when the lesions increase in severity, the transcription of L1 and L2 tends to disappear (9,18), which can cause false negatives that may be infected by high-risk virus types. Thus, some authors do not recommend IHC as a screening method for the diagnosis of HPV due to its low sensitivity (19). However, the problem could be the epitope to which the test is directed; antibodies directed to E6 or E7 could avoid this problem in infections by high-risk virus types.

The relatively low levels of detection of L1 by IHC can also be attributed to the antiquity of the samples, which influences the denaturation of L1; a low concentration of antibody used during the technique and the fixation and processing of tissues can have an adverse effect on the epitope (19).

In this study, we evaluated the effectiveness of IHC tests in identifying infections by HPV in the oral mucosa. Our results indicate that in people younger than 35±20.2 years, the expression of the L1 protein is more probable. This implies that in children and people younger than 35 years, there is a protective factor that prevents the methylation of the genes E1 and E2 in the URR region, avoiding the action of E6 and E7 and, hence, the integration of the viral DNA with the cellular genome, or may be that in people older than 35 years, there is a risk factor that causes the methylation of the E1 and E2 genes. Regarding this, Fernandez *et al*., reported that HPV genome does not encode for any DNA methyltransferases and thus it is believed that the viral genome is methylated by DNA methyltransferases of the human host cell (20). Balderas-Loaeza *et al*., demonstrated the methylation of the HPV16 genome near the 3’ end of the L1 ORF and in the URR, which regulates the expression of E6 and E7 through E2 (21) and Wilson *et al*., found that the sites of methylation within the viral genome are mainly detected at the boundary of the L1/L2 ORFs and within the E1 ORF (22). Thereby, HPV becomes subject of epigenetic modification (20).

The clinical presence of multiple lesions appears to represent a less aggressive HPV infection because it is associated with the expression of L1 and is more common in children and those younger than 35 years. An example is the MEH that is a lesion by HPV often presented as a multiple lesion mainly in children which frequently resolves spontaneously (23).

The presence of OC from infections by HPV where integration of the viral DNA to the host DNA does not exist suggests that the expression of E6 and E7 is not necessary for the development of cancer or these proteins can be activated without that integration. Concerning this, Rautava *et al*., explain that E6 and E7 of the low-risk HPV types can also bind to p53 and pRb respectively. In the case of p53 will not lead to its degradation but in the case of pRb the binding will have a lower affinity, nevertheless may lead to its transformation and it has been reported that E7 of the low-risk virus HPV1 can bind to pRb with the same affinity as E7 of HPV16 (13). The PCR test permits the identification of the presence (DNA) or expression of a gene (RNA) (24), whereas the IHC allows identifying the expression of a protein (25). Although the PCR is a powerful tool that enables the identification of the expression of genes, such expression can be affected by epigenetic alterations at the level of gene methylation, histone modification, and miRNA action during replication, transcription, and translation (26). Thus, their amplification and expression by PCR or IHC explains a probable tendency in a patient but do not guarantees the evolution or development of a lesion, and inconsistencies could be found. For this reason, their use should be accompanied by periodic clinical and histopathologic observations when a high-risk behavior is suspected.

## Conclusions

Although Toki’s criteria could be useful in the diagnoses of cervical lesions, they are not appropriate for the diagnosis of oral mucosal lesions by HPV through IHC.

We found clinical and histopathologic criteria that optimize the diagnosis of lesions by HPV in the oral mucosa through the identification of the L1 protein by IHC. However, they were few and have to be used prudently.
